# Bis{2-[2-(isopropyl­ammonio)ethyl­imino­meth­yl]-6-methoxy­phenolato}nickel(II) dithio­cyanate

**DOI:** 10.1107/S1600536808008052

**Published:** 2008-03-29

**Authors:** Hong-Bo Ma, Yan-Xia Jiang, Jun-Tao Lei

**Affiliations:** a Department of Nutrition, Jilin Medical College, Jilin 132013, People’s Republic of China; b Department of Biochemistry, Jilin Medical College, Jilin 132013, People’s Republic of China; c Department of Pharmacopedics, Jilin Medical College, Jilin 132013, People’s Republic of China

## Abstract

The title complex, [Ni(C_13_H_20_N_2_O_2_)_2_](NCS)_2_, consists of a centrosymmetric mononuclear four-coordinate nickel(II) complex cation and two thio­cyanate anions. The Ni atom is located on an inversion center and is coordinated by two phenol O atoms and two imine N atoms from two equivalent Schiff base ligands, in a square-planar geometry. In the crystal structure, the amino H atoms are involved in N—H⋯O hydrogen bonds with the phenol and meth­oxy O atoms of the ligand, and in N—H⋯N hydrogen bonds with the N atoms of the thio­cyanate anions, which sit above and below the Ni atom.

## Related literature

For background on the chemistry of Schiff base nickel(II) complexes, see: Marganian *et al.* (1995 ▶). For their biological activity, see: Harrop *et al.* (2003 ▶); Brückner *et al.* (2000 ▶); Ren *et al.* (2002 ▶). For thio­cyanate-coordinated complexes, see: Bogdanović *et al.* (2005 ▶); Schottenfeld *et al.* (2007 ▶); Abul-Haj *et al.* (2000 ▶). For related structures, see: Arıcı *et al.* (2005 ▶); Diao (2007 ▶); Diao *et al.* (2007 ▶); Zhu *et al.* (2004 ▶); Van Hecke *et al.* (2007 ▶); de Castro *et al.* (2001 ▶).
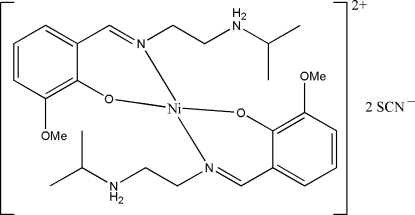


## Experimental

### 

#### Crystal data

[Ni(C_13_H_20_N_2_O_2_)_2_](NCS)_2_
*M* *_r_* = 647.49
Orthorhombic, 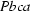

*a* = 13.520 (2) Å
*b* = 9.810 (3) Å
*c* = 24.102 (3) Å
*V* = 3196.7 (12) Å^3^
*Z* = 4
Mo *K*α radiation
μ = 0.78 mm^−1^
*T* = 298 (2) K
0.23 × 0.22 × 0.20 mm

#### Data collection

Bruker SMART CCD area-detector diffractometer
Absorption correction: multi-scan (*SADABS*; Bruker, 2001 ▶) *T*_min_ = 0.841, *T*_max_ = 0.860
24542 measured reflections
3863 independent reflections
1895 reflections with *I* > 2σ(*I*)
*R*_int_ = 0.110

#### Refinement

*R*[*F* ^2^ > 2σ(*F* ^2^)] = 0.070
*wR*(*F* ^2^) = 0.175
*S* = 1.01
3863 reflections
190 parameters
6 restraints
H-atom parameters constrained
Δρ_max_ = 0.29 e Å^−3^
Δρ_min_ = −0.38 e Å^−3^

### 

Data collection: *SMART* (Bruker, 2007 ▶); cell refinement: *SAINT* (Bruker, 2007 ▶); data reduction: *SAINT*; program(s) used to solve structure: *SHELXTL* (Sheldrick, 2008 ▶); program(s) used to refine structure: *SHELXTL*; molecular graphics: *SHELXTL*; software used to prepare material for publication: *SHELXTL*.

## Supplementary Material

Crystal structure: contains datablocks global, I. DOI: 10.1107/S1600536808008052/su2049sup1.cif

Structure factors: contains datablocks I. DOI: 10.1107/S1600536808008052/su2049Isup2.hkl

Additional supplementary materials:  crystallographic information; 3D view; checkCIF report

## Figures and Tables

**Table 1:** Hydrogen-bond geometry (Å, °)

*D*—H⋯*A*	*D*—H	H⋯*A*	*D*⋯*A*	*D*—H⋯*A*
N2—H2*B*⋯O2^i^	0.90	2.34	3.068 (5)	138
N2—H2*B*⋯O1^i^	0.90	1.88	2.664 (4)	145
N2—H2*A*⋯N3	0.90	2.13	2.983 (6)	158

Symmetry code: (i) 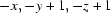.

**Fig. 1.**
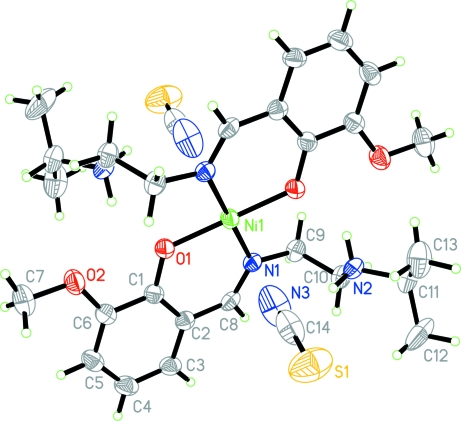
The molecular structure of complex (I), with displacement ellipsoids drawn at the 30% probability level.

## supplementary crystallographic information

### Comment

Nickel(II) complexes derived from Schiff bases have been widely studied (Marganian *et al.*, 1995). Some of them have been found to have pharmacological and antitumor properties (Harrop *et al.*, 2003; Brückner *et al.*, 2000; Ren *et al.*, 2002). The thiocyanate ligand displays a number of coordination modes and has become one of the most extensively studied building blocks in the synthesis of complexes (Bogdanović *et al.*, 2005; Schottenfeld *et al.*, 2007; Abul-Haj *et al.*, 2000), however, the thiocyanate group acting as a counterion in complexes has seldom been reported. We report herein the crystal structure of the title nickel(II) complex (I).

Complex (I) consists of a centrosymmetric mononuclear four-coordinated nickel(II) complex molecule and two thiocyanate anions (Fig. 1). The Ni atom is located on an inversion center and coordinated, by two phenolic O atoms and two imine N atoms from two equivalent Schiff base ligands, in a square planar geometry. The thiocyanate anions act as counterions and are not coordinate to the nickel(II) atom (Fig. 1). All the coordinate bond values are similar to those observed in other Schiff base nickel(II) complexes (Arıcı *et al.*, 2005; Diao, 2007; Diao *et al.*, 2007; Zhu *et al.*, 2004; Van Hecke *et al.*, 2007; de Castro *et al.*, 2001).

In the crystal structure of (I) the amino H-atoms are involved in N-H···O hydrogen bonds with the phenolic and methoxy O atoms of the ligand, and in N-H···N hydrogen bonds with the N-atom of the thiocyanate anions, which sit above and below the nickel atom (Table 1).

### Experimental

3-Methoxysalicylaldehyde (1.0 mmol, 152.0 mg), *N*-isopropylethane-1,2-diamine (1.0 mmol, 122.2 mg), ammonium thiocyanate (1.0 mmol, 76.0 mg), and Ni(NO_3_)_2_.6H_2_O (0.5 mmol, 145.0 mg) were dissolved in methanol (50 ml). The mixture was stirred at reflux for 2h to give a reddish solution. After keeping the solution in air for a few days, red block-like crystals were formed.

### Refinement

H atoms were positioned geometrically and refined using a riding model with d(N—H) = 0.90 Å, *U*_iso_ = 1.2*U*_eq_(N), and d(C—H) = 0.93 - 0.97 Å, *U*_iso_ = 1.2 or 1.5*U*_eq_ (C).

### Crystal data

**Table:**

[Ni(C_13_H_20_N_2_O_2_)_2_](NCS)_2_	*D*_x_ = 1.345 Mg m^−^^3^
*M**_r_* = 647.49	Mo *K*α radiation λ = 0.71073 Å
Orthorhombic, *P**b**c**a*	Cell parameters from 1440 reflections
*a* = 13.520 (2) Å	θ = 2.3–24.6º
*b* = 9.810 (3) Å	µ = 0.78 mm^−^^1^
*c* = 24.102 (3) Å	*T* = 298 (2) K
*V* = 3196.7 (12) Å^3^	Block, red
*Z* = 4	0.23 × 0.22 × 0.20 mm
*F*_000_ = 1368	

### Data collection

**Table:**

Bruker SMART CCD area-detector diffractometer	3863 independent reflections
Radiation source: fine-focus sealed tube	1895 reflections with *I* > 2σ(*I*)
Monochromator: graphite	*R*_int_ = 0.110
*T* = 298(2) K	θ_max_ = 28.3º
ω scans	θ_min_ = 1.7º
Absorption correction: multi-scan(SADABS; Bruker, 2001)	*h* = −17→17
*T*_min_ = 0.841, *T*_max_ = 0.860	*k* = −12→12
24542 measured reflections	*l* = −31→31

### Refinement

**Table:**

Refinement on *F*^2^	Secondary atom site location: difference Fourier map
Least-squares matrix: full	Hydrogen site location: inferred from neighbouring sites
*R*[*F*^2^ > 2σ(*F*^2^)] = 0.070	H-atom parameters constrained
*wR*(*F*^2^) = 0.175	*w* = 1/[σ^2^(*F*_o_^2^) + (0.0594*P*)^2^ + 2.0547*P*] where *P* = (*F*_o_^2^ + 2*F*_c_^2^)/3
*S* = 1.01	(Δ/σ)_max_ = 0.001
3863 reflections	Δρ_max_ = 0.29 e Å^−^^3^
190 parameters	Δρ_min_ = −0.38 e Å^−^^3^
6 restraints	Extinction correction: none
Primary atom site location: structure-invariant direct methods	

### Special details

**Table:**

Geometry. All e.s.d.'s (except the e.s.d. in the dihedral angle between two l.s. planes) are estimated using the full covariance matrix. The cell e.s.d.'s are taken into account individually in the estimation of e.s.d.'s in distances, angles and torsion angles; correlations between e.s.d.'s in cell parameters are only used when they are defined by crystal symmetry. An approximate (isotropic) treatment of cell e.s.d.'s is used for estimating e.s.d.'s involving l.s. planes.
Refinement. Refinement of *F*^2^ against ALL reflections. The weighted *R*-factor *wR* and goodness of fit *S* are based on *F*^2^, conventional *R*-factors *R* are based on *F*, with *F* set to zero for negative *F*^2^. The threshold expression of *F*^2^ > σ(*F*^2^) is used only for calculating *R*-factors(gt) *etc*. and is not relevant to the choice of reflections for refinement. *R*-factors based on *F*^2^ are statistically about twice as large as those based on *F*, and *R*- factors based on ALL data will be even larger.

### Fractional atomic coordinates and isotropic or equivalent isotropic displacement parameters (Å^2^)

**Table:**

	*x*	*y*	*z*	*U*_iso_*/*U*_eq_	
Ni1	0.0000	0.5000	0.5000	0.0503 (3)	
O1	0.0862 (2)	0.5589 (3)	0.55717 (11)	0.0595 (8)	
O2	0.1713 (2)	0.7110 (3)	0.62919 (13)	0.0679 (9)	
S1	0.12952 (16)	0.8799 (3)	0.34887 (10)	0.1446 (9)	
N1	0.1017 (2)	0.3634 (3)	0.47434 (13)	0.0462 (8)	
N2	0.0395 (3)	0.4013 (3)	0.35925 (13)	0.0511 (9)	
H2A	0.0740	0.4743	0.3710	0.061*	
H2B	−0.0197	0.4034	0.3763	0.061*	
N3	0.1018 (5)	0.6596 (5)	0.4145 (3)	0.123 (2)	
C1	0.1838 (3)	0.5537 (4)	0.55731 (16)	0.0479 (10)	
C2	0.2397 (3)	0.4670 (4)	0.52411 (17)	0.0474 (10)	
C3	0.3429 (3)	0.4673 (5)	0.5277 (2)	0.0566 (12)	
H3	0.3799	0.4086	0.5056	0.068*	
C4	0.3893 (3)	0.5529 (5)	0.5634 (2)	0.0659 (13)	
H4	0.4581	0.5543	0.5648	0.079*	
C5	0.3353 (3)	0.6389 (5)	0.59804 (19)	0.0610 (13)	
H5	0.3678	0.6967	0.6226	0.073*	
C6	0.2337 (3)	0.6377 (4)	0.59571 (17)	0.0523 (11)	
C7	0.2135 (4)	0.8054 (5)	0.6670 (2)	0.0850 (16)	
H7A	0.2530	0.7573	0.6937	0.127*	
H7B	0.1617	0.8540	0.6857	0.127*	
H7C	0.2544	0.8687	0.6471	0.127*	
C8	0.1937 (3)	0.3693 (4)	0.48742 (15)	0.0493 (11)	
H8	0.2349	0.3040	0.4717	0.059*	
C9	0.0744 (3)	0.2481 (4)	0.43785 (17)	0.0553 (11)	
H9A	0.0050	0.2267	0.4433	0.066*	
H9B	0.1126	0.1686	0.4484	0.066*	
C10	0.0920 (3)	0.2777 (4)	0.37715 (17)	0.0560 (11)	
H10A	0.1623	0.2892	0.3707	0.067*	
H10B	0.0697	0.2009	0.3551	0.067*	
C11	0.0231 (4)	0.4150 (5)	0.29841 (17)	0.0681 (14)	
H11	−0.0102	0.3323	0.2853	0.082*	
C12	0.1198 (4)	0.4270 (7)	0.2686 (2)	0.119 (2)	
H12A	0.1559	0.5037	0.2829	0.178*	
H12B	0.1080	0.4397	0.2297	0.178*	
H12C	0.1577	0.3453	0.2741	0.178*	
C13	−0.0449 (5)	0.5349 (6)	0.2881 (2)	0.0964 (19)	
H13A	−0.1083	0.5172	0.3045	0.145*	
H13B	−0.0526	0.5481	0.2489	0.145*	
H13C	−0.0169	0.6155	0.3043	0.145*	
C14	0.1096 (5)	0.7465 (7)	0.3872 (3)	0.095 (2)	

### Atomic displacement parameters (Å^2^)

**Table:**

	*U*^11^	*U*^22^	*U*^33^	*U*^12^	*U*^13^	*U*^23^
Ni1	0.0405 (4)	0.0602 (5)	0.0501 (4)	0.0056 (4)	−0.0042 (4)	−0.0156 (4)
O1	0.0406 (17)	0.084 (2)	0.0535 (18)	0.0023 (15)	0.0001 (14)	−0.0189 (16)
O2	0.066 (2)	0.069 (2)	0.068 (2)	−0.0026 (17)	−0.0098 (17)	−0.0192 (18)
S1	0.1218 (16)	0.166 (2)	0.1462 (18)	0.0331 (15)	0.0288 (14)	0.0599 (16)
N1	0.049 (2)	0.049 (2)	0.0408 (19)	−0.0021 (17)	0.0034 (16)	0.0038 (16)
N2	0.057 (2)	0.052 (2)	0.045 (2)	0.0072 (18)	0.0093 (17)	−0.0007 (17)
N3	0.162 (5)	0.062 (3)	0.147 (5)	0.011 (3)	−0.065 (4)	0.008 (3)
C1	0.046 (3)	0.053 (2)	0.045 (2)	−0.002 (2)	−0.004 (2)	0.008 (2)
C2	0.049 (3)	0.050 (3)	0.043 (2)	0.004 (2)	0.001 (2)	0.0088 (19)
C3	0.042 (3)	0.067 (3)	0.060 (3)	0.004 (2)	0.002 (2)	0.007 (2)
C4	0.045 (3)	0.082 (3)	0.071 (3)	−0.003 (3)	−0.006 (2)	0.014 (3)
C5	0.061 (3)	0.063 (3)	0.059 (3)	−0.015 (2)	−0.016 (2)	0.017 (2)
C6	0.052 (3)	0.052 (3)	0.053 (3)	−0.003 (2)	−0.005 (2)	0.008 (2)
C7	0.107 (4)	0.069 (3)	0.079 (4)	−0.008 (3)	−0.023 (3)	−0.013 (3)
C8	0.054 (3)	0.056 (3)	0.038 (2)	0.011 (2)	0.0101 (19)	0.0086 (19)
C9	0.071 (3)	0.045 (2)	0.051 (3)	0.004 (2)	0.003 (2)	−0.001 (2)
C10	0.070 (3)	0.048 (3)	0.050 (3)	0.012 (2)	0.000 (2)	−0.004 (2)
C11	0.095 (4)	0.065 (3)	0.044 (3)	0.010 (3)	−0.001 (3)	−0.001 (2)
C12	0.141 (6)	0.157 (6)	0.058 (4)	0.035 (5)	0.041 (4)	0.028 (4)
C13	0.137 (5)	0.080 (4)	0.072 (4)	0.032 (4)	−0.025 (4)	0.007 (3)
C14	0.095 (4)	0.075 (4)	0.116 (5)	0.015 (4)	−0.039 (4)	−0.022 (4)

### Geometric parameters (Å, °)

**Table:**

Ni1—O1^i^	1.895 (3)	C4—H4	0.9300
Ni1—O1	1.895 (3)	C5—C6	1.374 (6)
Ni1—N1^i^	2.017 (3)	C5—H5	0.9300
Ni1—N1	2.017 (3)	C7—H7A	0.9600
O1—C1	1.320 (5)	C7—H7B	0.9600
O2—C6	1.371 (5)	C7—H7C	0.9600
O2—C7	1.418 (5)	C8—H8	0.9300
S1—C14	1.624 (8)	C9—C10	1.511 (5)
N1—C8	1.285 (5)	C9—H9A	0.9700
N1—C9	1.479 (5)	C9—H9B	0.9700
N2—C10	1.470 (5)	C10—H10A	0.9700
N2—C11	1.489 (5)	C10—H10B	0.9700
N2—H2A	0.9000	C11—C12	1.497 (7)
N2—H2B	0.9000	C11—C13	1.513 (7)
N3—C14	1.082 (7)	C11—H11	0.9800
C1—C2	1.391 (6)	C12—H12A	0.9600
C1—C6	1.412 (5)	C12—H12B	0.9600
C2—C3	1.399 (6)	C12—H12C	0.9600
C2—C8	1.445 (6)	C13—H13A	0.9600
C3—C4	1.356 (6)	C13—H13B	0.9600
C3—H3	0.9300	C13—H13C	0.9600
C4—C5	1.393 (6)		
			
O1^i^—Ni1—O1	180.000 (1)	O2—C7—H7C	109.5
O1^i^—Ni1—N1^i^	90.38 (13)	H7A—C7—H7C	109.5
O1—Ni1—N1^i^	89.62 (13)	H7B—C7—H7C	109.5
O1^i^—Ni1—N1	89.62 (13)	N1—C8—C2	126.7 (4)
O1—Ni1—N1	90.38 (13)	N1—C8—H8	116.7
N1^i^—Ni1—N1	180.00 (17)	C2—C8—H8	116.7
C1—O1—Ni1	127.2 (3)	N1—C9—C10	112.9 (3)
C6—O2—C7	118.2 (4)	N1—C9—H9A	109.0
C8—N1—C9	115.0 (4)	C10—C9—H9A	109.0
C8—N1—Ni1	123.7 (3)	N1—C9—H9B	109.0
C9—N1—Ni1	121.4 (3)	C10—C9—H9B	109.0
C10—N2—C11	115.8 (3)	H9A—C9—H9B	107.8
C10—N2—H2A	108.3	N2—C10—C9	111.5 (3)
C11—N2—H2A	108.3	N2—C10—H10A	109.3
C10—N2—H2B	108.3	C9—C10—H10A	109.3
C11—N2—H2B	108.3	N2—C10—H10B	109.3
H2A—N2—H2B	107.4	C9—C10—H10B	109.3
O1—C1—C2	124.4 (4)	H10A—C10—H10B	108.0
O1—C1—C6	117.2 (4)	N2—C11—C12	110.4 (4)
C2—C1—C6	118.3 (4)	N2—C11—C13	108.8 (4)
C1—C2—C3	120.3 (4)	C12—C11—C13	113.0 (5)
C1—C2—C8	121.6 (4)	N2—C11—H11	108.2
C3—C2—C8	118.0 (4)	C12—C11—H11	108.2
C4—C3—C2	120.2 (4)	C13—C11—H11	108.2
C4—C3—H3	119.9	C11—C12—H12A	109.5
C2—C3—H3	119.9	C11—C12—H12B	109.5
C3—C4—C5	120.8 (4)	H12A—C12—H12B	109.5
C3—C4—H4	119.6	C11—C12—H12C	109.5
C5—C4—H4	119.6	H12A—C12—H12C	109.5
C6—C5—C4	119.6 (4)	H12B—C12—H12C	109.5
C6—C5—H5	120.2	C11—C13—H13A	109.5
C4—C5—H5	120.2	C11—C13—H13B	109.5
O2—C6—C5	125.9 (4)	H13A—C13—H13B	109.5
O2—C6—C1	113.4 (4)	C11—C13—H13C	109.5
C5—C6—C1	120.7 (4)	H13A—C13—H13C	109.5
O2—C7—H7A	109.5	H13B—C13—H13C	109.5
O2—C7—H7B	109.5	N3—C14—S1	175.4 (7)
H7A—C7—H7B	109.5		

Symmetry codes: (i) −*x*, −*y*+1, −*z*+1.

### Hydrogen-bond geometry (Å, °)

**Table:**

*D*—H···*A*	*D*—H	H···*A*	*D*···*A*	*D*—H···*A*
N2—H2B···O2^i^	0.90	2.34	3.068 (5)	138
N2—H2B···O1^i^	0.90	1.88	2.664 (4)	145
N2—H2A···N3	0.90	2.13	2.983 (6)	158

Symmetry codes: (i) −*x*, −*y*+1, −*z*+1.
